# Joint Associations of Maternal Gestational Diabetes and Hypertensive Disorders of Pregnancy With Overweight in Offspring

**DOI:** 10.3389/fendo.2019.00645

**Published:** 2019-09-20

**Authors:** Yuying Gu, Jun Lu, Weiqin Li, Huikun Liu, Leishen Wang, Junhong Leng, Wei Li, Shuang Zhang, Shuting Wang, Jaakko Tuomilehto, Zhijie Yu, Xilin Yang, Andrea A. Baccarelli, Lifang Hou, Gang Hu

**Affiliations:** ^1^Chronic Disease Epidemiology Laboratory, Pennington Biomedical Research Center, Baton Rouge, LA, United States; ^2^Shanghai Business School, Shanghai, China; ^3^Department of Endocrinology and Metabolism, Fengxian Hospital of Southern Medical University, Shanghai, China; ^4^Tianjin Women's and Children's Health Center, Tianjin, China; ^5^Department of Public Health, University of Helsinki, Helsinki, Finland; ^6^Population Cancer Research Program, Dalhousie University, Halifax, NS, Canada; ^7^Department of Epidemiology, School of Public Health, Tianjin Medical University, Tianjin, China; ^8^Department of Environmental Health Sciences, Columbia University Mailman School of Public Health, New York, NY, United States; ^9^Department of Preventive Medicine, Feinberg School of Medicine, Northwestern University, Chicago, IL, United States

**Keywords:** gestational diabetes mellitus, hypertensive disorders of pregnancy, overweight, obesity, childhood

## Abstract

**Objectives:** Either maternal gestational diabetes mellitus (GDM) or hypertensive disorder of pregnancy (HDP) is associated with an increased risk of obesity in the offspring. However, their joint associations with obesity in offspring remain unclear. We investigated the joint associations of maternal GDM and HDP with childhood overweight in offspring.

**Methods:** We performed a large study in 1967 mother-child pairs. Maternal GDM was diagnosed according to the 1999 World Health Organization (WHO) criteria. HDP was defined as self-reported doctor-diagnosed hypertension or treatment of hypertension (including gestational hypertension, preeclampsia, sever preeclampsia or eclampsia) after 20 weeks of gestation on the questionnaire. Body mass index (BMI) for age Z-score and childhood overweight were evaluated according to WHO growth reference. We used the general linear models to compare children's Z score for BMI and logistic regression models to estimate odds ratios of childhood overweight according to maternal different status of GDM and HDP.

**Results:** Offspring of mothers with both GDM and HDP had a higher BMI for age Z-score (0.63 vs. 0.03, *P* < 0.001) than children born to normotensive and normoglycemic pregnancy. After adjustment for maternal and children's major confounding factors, joint GDM and HDP were associated with increased odds ratios of offspring's overweight compared with normotensive and normoglycemic pregnancy (2.97, 95% confidence intervals [CIs] 1.65–5.34) and GDM alone (2.06, 95% CIs 1.20–3.54), respectively. After additional adjustment for maternal pre-pregnancy BMI and gestational weight gain, joint maternal GDM, and HDP was still associated with an increased risk of offspring's overweight compared with the maternal normotensive, and normoglycemic group but became to have a borderline increased risk compared with the maternal GDM alone group.

**Conclusions:** Maternal GDM alone or joint GDM and HDP were associated with increased ratios of offspring's overweight.

## Introduction

Children with overweight and obesity are more likely to be affected by obesity into adulthood and develop non-communicable diseases including metabolic syndrome, cardiovascular diseases, and type 2 diabetes (1–3). The prevalence of obesity among children has been increasing globally in the past 25 years (4). In China, the prevalence of overweight and obesity among preschoolers reached 10.1% in 2010, and obesity was much worse in children than in adolescents (5, 6). China now has the largest number of children with obesity in the world (4).

Changes in children's behaviors, such as unhealthy eating habit, lacking moderate-to-vigorous physical activity, and increasing sedentary time, mainly account for the increasing prevalence of childhood obesity (7–9). Other factors, such as genetic susceptibility, socioeconomic inequalities, maternal education, children's sleep disturbance, neonatal macrosomia, may also play an important role in obesity formation (8–11). Recent studies have indicated that maternal metabolic disorders during pregnancy, such as gestational diabetes mellitus (GDM), and hypertensive disorder of pregnancy (HDP), were associated with an increased risk of obesity in children over 5 years old. Several but not all studies have found that maternal GDM was positively associated with general overweight/obesity, central obesity, and subcutaneous adiposity (skinfold thickness) in the offspring after 5 years old (12–17). Meanwhile, researchers pointed out that maternal HDP was associated with a greater risk of childhood overweight or obesity (18–20). A previous study from our team also demonstrated that maternal HDP was a risk factor for childhood overweight or obesity in the offspring of mothers with GDM (21). Nevertheless, it is unclear whether GDM concomitant with HDP represents an extra higher risk factor for obesity in offspring. We aimed to examine the joint association of maternal GDM and HDP with children's overweight among 1,967 mother-child pairs, including 1,263 mothers with GDM, and 704 without GDM.

## Materials and Methods

### GDM Screening Process

Tianjin is the fourth largest city in China, only 30-min distance by train from Beijing. There are six central districts in Tianjin with about 4.3 million residents. In 1999, the Tianjin Women's and Children's Health Center launched an urban universal screening of GDM using the 1999 World Health Organization (WHO)'s criteria in all six central districts. The screening rate was reported to be >91% between 1999 and 2008 (22). We first invited all pregnant women (at their 26–30 gestational weeks) to participate in the 1-h 50-g glucose screening test in their community health centers. Then, those with glucose reading ≥7.8 mmol/L were referred to the Tianjin Women's and Children's Health Center to undergo a 2-h oral glucose tolerance test (OGTT) with 75-g glucose load. If the pregnant women met the 1999 WHO's criteria of diabetes (fasting glucose ≥7 mmol/L or 2-h glucose ≥11.1 mmol/L), or impaired glucose tolerance (IGT) (2-h glucose ≥7.8 mmol/L and <11.1 mmol/L), they would be diagnosed as GDM (23).

### Study Population

Totally 76,325 women were screened from 2005 to 2009, among whom 4,644 women were diagnosed as GDM, and 71,681 were free of GDM. We invited all 4,644 women with GDM to participate in the Tianjin Gestational Diabetes Mellitus Prevention Program (TGDMPP). From August 2009 to July 2011, a total of 1,263 women with GDM and their children finished the baseline survey. There were no differences at 26–30 gestational weeks OGTT test in age (28.9 vs. 28.7 years), fasting glucose (5.34 vs. 5.34 mmol/l), 2-h glucose (9.23 vs. 9.16 mmol/l), and the prevalence of IGT (90.9 vs. 91.8%) and diabetes (9.1 vs. 8.2%) between the returned and unreturned GDM women. We randomly chose and recruited 704 non-GDM mother-child pairs, with age and sex frequency-matched to children of GDM mothers at 1–2 years after the baseline survey of GDM mother-child pairs.

This study was carried out in accordance with the recommendations of the Human Subjects Committee of Tianjin Women's and Children's Health Center with written informed consent from all subjects. All subjects gave written informed consent in accordance with the Declaration of Helsinki. The protocol was approved by the Human Subjects Committee of Tianjin Women's and Children's Health Center.

### Questionnaires and Measurements

A self-administered questionnaire was implemented to collect mothers' information, including socio-demographic characteristics, such as age, education (<13 years, 13–16 years, and ≥16 years), history of GDM and its treatment during pregnancy; pregnancy outcomes (pre-pregnancy weight, weight gain during pregnancy, gestational age, etc.); and lifestyle in the past year, such as smoking status. HDP was defined as self-reported doctor-diagnosed hypertension or treatment of hypertension (including gestational hypertension, preeclampsia, sever preeclampsia or eclampsia) after 20 weeks of gestation on the questionnaire (8). Children's information was collected by another questionnaire completed by their mothers, including children's general information, such as sex, birth date, age, birth weight, birth length, lactation (exclusive formula, mixed, or exclusive breast) and lactation duration; history of diseases, and medication; dietary habits [using a validated food frequency questionnaire [FFQ]] (24); and routine activities (indoor and outdoor activities, screening watching time, and sleep duration) (25). Macrosomia was defined when birth weight ≥4,000 g.

All mother-child pairs underwent a physical examination. Using the standardized protocol, all participants' height and weight were measured in light indoor clothing and without shoes by trained research doctors. Body mass index (BMI) was obtained by dividing weight in kilograms by the square of height in meters. All mothers' pre-pregnancy BMI used their self-reported pre-pregnancy weight and their measured height. Children's BMI calculation used their body weight and height examined in the study visit. Children's Z scores for BMI-for-age were calculated based on the WHO growth reference (26, 27). Children's BMI was classified as normal weight, BMI <85th percentiles; overweight, 85th percentile ≤ BMI <95th percentile; and obesity, BMI ≥95th percentile, according to the WHO age-, and sex-specific growth reference (26, 27).

### Statistical Analysis

Differences in the general characteristics (continuous and categorical variables) of both mothers and children according to maternal different status of GDM and HDP were tested using the univariate analysis of variance or chi-square test. We used the general linear models.

to compare children's Z scores for BMI and used logistic regression models to estimate odds ratios of childhood overweight according to maternal different status of GDM and HDP. All analyses were adjusted for maternal age, gestational age, education, smoking status, history of treatment of GDM, and HDP, children's sex, age, birth weight, and feeding status (Model 1), and then children's lifestyles including outdoor physical activity time, screen watching time, sleep time, daily energy intake, energy from fat and dietary fiber intake (Model 2), and further for maternal pre-pregnancy BMI and gestational weight gain (Model 3). All the statistical analyses were performed with SPSS 25.0 for windows software package (IBM SPSS statistics 25) and SAS Proprietary Software 9.4 (SAS Institute Inc., Cary, NC, USA). Two-sided *P* < 0.05 was considered statistically significant.

## Results

As shown in Table 1, there were significant differences in maternal age at delivery, pre-pregnancy BMI, gestational weight gain, gestational age at delivery, and education among 4 different groups (all *P* < 0.05). There were also differences in children's age, birth weight, outdoor activity time, screen watching time, Z score for BMI-for-age, prevalence of overweight/obesity, and daily energy intake from fat according to various maternal GDM and HDP status (*P* < 0.001).

**Table 1:** Maternal and child characteristics according to maternal gestational diabetes and hypertensive disorders of pregnancy.

	**Non-GDM**		**GDM**		***P*-value**
	**Non-HDP**	**HDP**	**Non-HDP**	**HDP**	
Numbers of subjects	677	27	1172	91	
**Maternal characteristics**					
Age at delivery, years	32.9 ± 2.84	32.9 ± 2.68	33.3 ± 3.49	33.8 ± 3.59	0.032
Pre-pregnancy BMI, kg/m^2^	21.4 ± 2.96	22.2 ± 3.04	22.9 ± 3.24	25.1 ± 3.64	< 0.001
Gestational weight gain, kg	18.2 ± 6.67	21.3 ± 6.14	16.6 ± 5.87	19.0 ± 7.01	< 0.001
Gestational age at delivery, weeks	39.1 ± 1.48	38.4 ± 1.76	39.0 ± 1.48	38.6 ± 1.77	0.001
Education, %					< 0.001
<13 years	10.6	3.7	21.9	29.7	
13–16 years	74.9	88.9	70.5	64.8	
≥16 years	14.5	7.4	7.6	5.5	
Current smoker, %	4.1	0	1.9	3.3	0.011
Insulin treatment of GDM, %	-	-	4.4	5.5	< 0.001
Anti-hypertensive therapy of HDP, %	-	40.7	-	28.6	< 0.001
**Child characteristics**					
Boy, %	52.0	63.0	52.3	62.6	0.43
Age, years	5.25 ± 1.20	5.41 ± 1.31	4.60 ± 2.18	4.23 ± 2.24	< 0.001
Birth weight, gram	3,425 ± 449	3,080 ± 583	3,540 ± 517	3,619 ± 613	< 0.001
Macrosomia	11.2	7.4	19.1	28.6	< 0.001
Mode of infant feeding, %					0.40
Exclusive breastfeeding	42.7	29.6	44.1	27.5	
Exclusive formula feeding	43.7	59.3	42.1	49.5	
Mixed feeding	13.6	11.1	13.8	23.1	
Outdoor activity, hours/day	1.04 ± 0.63	0.98 ± 0.54	1.41 ± 0.79	1.41 ± 0.90	< 0.001
Screen watching time, hours/day	0.93 ± 0.76	0.96 ± 0.73	1.13 ± 0.87	1.35 ± 1.05	< 0.001
Sleeping time, %					0.53
≤ 8 h/day	10.3	3.7	12.5	20.9	
9–10 h/day	66.8	81.5	58.8	52.7	
≥11 h/day	22.9	14.8	28.7	26.4	
BMI, kg/m^2^	15.6 ± 2.13	16.5 ± 3.51	16.2 ± 2.43	17.0 ± 2.93	< 0.001
Z score for BMI-for-age	−0.01 ± 1.23	0.43 ± 1.68	0.28 ± 1.28	0.77 ± 1.66	< 0.001
Prevalence of overweight/obesity, %	17.0	25.9	23.8	36.3	< 0.001
Energy intake, kcal/day	1,526 ± 424	1,688 ± 563	1,259 ± 568	1,220 ± 612	< 0.001
Energy intake from fat, %	25.7 ± 5.40	25.8 ± 5.99	32.4 ± 21.7	33.1 ± 20.5	< 0.001
Diet fiber, gram/1,000 kcal	5.96 ± 1.56	5.76 ± 1.39	4.48 ± 1.87	4.22 ± 1.82	< 0.001

*BMI, body mass index; GDM, gestational diabetes mellitus; HDP, hypertensive disorders of pregnancy*.

*BMI for age Z-score and overweight/obesity in children were evaluated according to the age- and sex-specific growth reference issued by World Health Organization*.

In comparison with children born to normotensive and normoglycemic pregnancy, offspring of mothers with HDP alone, GDM alone, and concomitant GDM and HDP had higher levels of Z score for BMI-for-age after adjustment for maternal age at delivery, gestational age at delivery, education, current smoking, history of treatment of GDM and HDP, and children's age, sex, birth weight, and feeding status (Model 1, Table 2). After further adjustment for children's lifestyle factors, maternal pre-pregnancy BMI, and gestational weight gain, the difference of Z score for BMI was still significant among children who born to mothers with GDM alone and concomitant GDM and HDP, but no longer significant among children born to HDP pregnancy alone, compared with those born to normotensive and normoglycemic mothers during pregnancy.

**Table 2:** Comparison of BMI for age Z-score according to maternal gestational diabetes and hypertensive disorders of pregnancy.

	**Non-GDM**		**GDM**		***P*-value**
	**Non-HDP**	**HDP**	**Non-HDP**	**HDP**	
No. of subjects	677	27	1172	91	
**BMI for age Z-score**	0.03 ± 0.05	0.55 ± 0.26^*^	0.27 ± 0.04^†^	0.63 ± 0.15^†^^‡^	<0.001
Model 1							
Model 2	0.03 ± 0.05	0.43 ± 0.25	0.27 ± 0.04^†^	0.60 ± 0.14^†^^‡^	<0.001
Model 3	0.05 ± 0.05	0.30 ± 0.25	0.28 ± 0.04^†^	0.46 ± 0.14^*^	0.002

*Data were estimated means with SE or otherwise indicated*.

GDM, gestational diabetes mellitus; HDP, hypertensive disorders of pregnancy.

* *P < 0.05 compared with non-GDM and non-HDP group*.

† *P < 0.001 compared with non-GDM and non-HDP group*.

‡ *P < 0.05 compared with GDM and non-HDP group*.

*Model 1: adjusted for maternal age at delivery, gestational age at delivery, education, current smoking, history of insulin treatment of GDM, history of anti-hypertensive treatment of HDP, and children's age, sex, birth weight, and feeding status*.

*Model 2: adjusted for variables in Model 1 plus children's outdoor physical activity time, screen-watching time, sleep time, daily energy intake, dietary fiber intake and energy intake from fat*.

*Model 3: adjusted for variables in Model 2 plus maternal pre-pregnancy BMI and gestational weight gain*.

Compared with children born to normotensive and normoglycemic pregnancy, multi-variable (maternal age, gestational age, education and smoking status, history of treatment of GDM, and HDP, children's sex, age, birth weight, and feeding status) adjusted odds ratios (95% confidence intervals [95% CI]) of overweight among children who born to mothers with HDP alone, with GDM only, and with both GDM and HDP were 2.61 (95% CI 0.98–6.93), 1.51 (95% CI 1.17–1.95), and 3.11 (95% CI 1.78–5.43), respectively (Model 1, Table 3). After additional adjustment for children's lifestyle factors, maternal pre-pregnancy BMI, and gestational weight gain, children who born to mothers with GDM only or with both GDM and HDP still had significantly increased odds ratios of overweight compared with children who born to mothers with normotension and normoglycaemia during pregnancy (Model 2-3, Table 3). There was no difference in odds ratios of childhood overweight between children who born to mothers with HDP only and children who born to mothers with GDM only. Children who were born to mothers with joint GDM and HDP had ~1-fold higher multivariable-adjusted risk of overweight compared with children who were born to mothers with GDM only, but this association changed to borderline significance after additional adjustment for maternal pre-pregnancy BMI and gestational weight gain.

**Table 3:** Odds ratios for childhood overweight according to maternal gestational diabetes and hypertensive disorders of pregnancy.

	**Odds ratios (95% confidence intervals)**				***P* for trend**
	**Non-GDM**		**GDM**		
	**Non-HDP**	**HDP**	**Non-HDP**	**HDP**	
No. of participants	677	27	1172	91	
No. of cases	115	7	279	33	
Multivariable adjusted model 1	1.00	2.61 (0.98–6.93)	1.51 (1.17–1.95)	3.11 (1.78–5.43)	<0.001
Multivariable adjusted model 2	1.00	2.13 (0.75–6.01)	1.48 (1.11–1.96)	2.97 (1.65–5.34)	0.001
Multivariable adjusted model 3	1.00	1.67 (0.57–4.94)	1.46 (1.09–1.96)	2.34 (1.28–4.27)	0.003
**Additional analyses**					
Multivariable adjusted model 1		1.67 (0.63–4.42)	1	2.09 (1.23–3.54)	0.006
Multivariable adjusted model 2		1.39 (0.49–3.99)	1	2.06 (1.20–3.54)	0.009
Multivariable adjusted model 3		1.14 (0.38–3.45)	1	1.66 (0.95–2.90)	0.079

*GDM, gestational diabetes mellitus; HDP, hypertensive disorders of pregnancy*.

*Overweight/obesity were defined as body mass index ≥85th percentile according to the age-, and sex-specific world health organization reference range in Children*.

Model 1: adjusted for maternal age at delivery, gestational age at delivery, education, current smoking, history of insulin treatment of GDM, history of anti-hypertensive treatment of HDP, and children's age, sex, birth weight, and feeding status.

Model 2: adjusted for variables in Model 1 plus children's outdoor physical activity time, screen-watching time, sleep time, daily energy intake, dietary fiber intake and energy intake from fat.

*Model 3: adjusted for variables in Model 2 plus maternal pre-pregnancy BMI and gestational weight gain*.

## Discussion

The present study indicated that maternal GDM alone or joint GDM and HDP was associated with an increased Z score for BMI-for-age and a higher risk of childhood overweight in offspring. Concomitant of maternal GDM and HDP showed an additionally higher risk for offspring's overweight than maternal GDM alone in offspring, but this association was not fully independent of maternal pre-pregnancy BMI and gestational weight gain.

Maternal GDM and HDP are two common complications during pregnancy, and are associated with adverse pregnant outcomes and exert long-term effect on their children (12, 28). Most but not all studies have indicated that children born to mothers with GDM are at higher risk of overweight or obesity compared with those born to mothers with normal glucose during pregnancy (15, 29–31). Studies on maternal HDP with children's obesity were controversial. Most studies demonstrated that maternal HDP was associated with elevated BMI or an increased risk for children's overweight and obesity (18–21). However, a US study showed that offspring of mothers with HDP had a reduced Z score for BMI compared with children of non-HDP mothers (32). This study also showed that children born to mothers with GDM alone had a 1.5-fold increased risk for overweight/obesity compared with children born to mothers with normotension and normoglycaemia during pregnancy, and there was no difference in odd ratio of overweight/obesity between children born to mothers with GDM alone and those born to mothers with HDP alone. Furthermore, although birth weight was a risk factor for childhood obesity (8), children born to HDP pregnancy had a lower or similar birth weight when compared with the controls (18, 19, 32), implying the association between maternal HDP and children's obesity was complicated and long lasting. Until now, very few studies have assessed the joint association of maternal GDM and HDP with the childhood obesity, and results remained unknown. Kvehaugen and colleagues reported a non-significant higher proportion of overweight or obesity in children of mothers with GDM or preeclampsia than children of mothers without GDM and HDP (33). The present study showed that children born to mothers with joint GDM and HDP were associated with higher odds ratios for overweight/obesity than those born to mothers with normotension and normoglycaemia during pregnancy, and with GDM alone.

The mechanism for the association of maternal GDM and HDP with children's overweight and obesity has not been fully understood. Shared familial genes and lifestyle may be the first consideration as the reason of this association (20). However, some researchers ascribed the mechanism to intrauterine programming of epigenome. Data indicated that siblings who were born to uncomplicated pregnancy had significantly lower prevalence of obesity and vascular abnormalities than those born to HDP- or GDM- complicated pregnancy (34, 35). Some studies also demonstrated that placental gene-specific hypomethylation occurred more often in early-onset than late-onset pre-eclampsia (36, 37). Further, amelioration of maternal GDM can help reduce the odds ratio for children's obesity (12). All these clues supported intrauterine programming of epigenome in the fetus, and implied the importance of early prevention, management and treatment of metabolic disorder for next generation's health.

Insulin resistance may mediate GDM and HDP with intrauterine programming of epigenome, because insulin resistance was putative as pathogenesis of HDP and GDM in pregnant women (38–41), and cord plasma insulin level was positively associated with birth weight and neonatal fat mass (42). We postulate that the combination of GDM and HDP may imply more severe insulin resistance, as women with both GDM and HDP had the higher level of pre-pregnancy BMI, a parameter reflecting insulin resistance. This point of view needs further investigation.

Our study enrolled a larger sample of GDM and non-GDM mother-child pairs than previous studies. Data on a variety of confounding variables, such as the parameters of mothers before and during pregnancy; and indices of the children, including birth weight, lifestyles factors, and anthropometric indexes were collected and used in the final analysis. There were some limitations in our study. First, maternal HDP, and children's diet intake, physical activity and sleeping time were based on the self-reported questionnaire, which may bring retrospective bias. However, good concordance between self-reported HDP and clinical records has been validated in the United States and England (43). Second, the sample size in only HDP group was relatively small. Third, other confounders of children's obesity, such as the genetics, insulin resistance, have not been investigated in the present study.

In conclusion, the present study indicated that maternal GDM alone or joint GDM and HDP was associated with an increased Z score for BMI-for-age and a higher risk of childhood overweight in offspring. Maternal GDM and HDP had similar odds ratio for children's overweight. Joint maternal GDM and HDP were associated with extra higher odds ratios of offspring's overweight than maternal GDM alone but this association was not fully independent of maternal pre-pregnancy BMI and gestational weight gain.

## Data Availability Statement

All datasets generated for this study are included in the manuscript/Supplementary Files.

## Ethics Statement

This study was carried out in accordance with the recommendations of the Human Subjects Committee of Tianjin Women's and Children's Health Center with written informed consent from all subjects. All subjects gave written informed consent in accordance with the Declaration of Helsinki. The protocol was approved by the Human Subjects Committee of Tianjin Women's and Children's Health Center.

## Author Contributions

YG, JLu, and GH conceptualized and designed the study, performed statistical analyses, interpreted the results, and drafted and revised the manuscript. WqL, HL, LW, JLe, WL, SZ, and SW collected the data and revised the manuscript. JT, ZY, XY, AB, and LH critically revised the manuscript for important intellectual contents. All authors critically reviewed the scientific content and approved the final manuscript. GH is the guarantor of this work, and has full access to all the data in the study and takes responsibility for the integrity of the data and the accuracy of the data analysis.

### Conflict of Interest

The authors declare that the research was conducted in the absence of any commercial or financial relationships that could be construed as a potential conflict of interest. The reviewers and handling editor declared their shared affiliation.
